# Document analysis in health policy research: the READ approach

**DOI:** 10.1093/heapol/czaa064

**Published:** 2020-11-11

**Authors:** Sarah L Dalglish, Hina Khalid, Shannon A McMahon

**Affiliations:** 1 Department of International Health, Johns Hopkins School of Public Health, 615 N. Wolfe St, Baltimore, MD 21205, USA; 2 Institute for Global Health, University College London, Institute for Global Health 3rd floor, 30 Guilford Street, London WC1N 1EH, UK; 3 School of Humanities and Social Sciences, Information Technology University, Arfa Software Technology Park, Ferozepur Road, Lahore 54000, Pakistan; 4 Heidelberg Institute of Global Health, Medical Faculty and University Hospital, University of Heidelberg, Im Neuenheimer Feld 130/3, 69120 Heidelberg, Germany

**Keywords:** Health policy, health systems research, interdisciplinary, methods, policy, policy analysis, policy research, qualitative, research methods, social sciences

## Abstract

Document analysis is one of the most commonly used and powerful methods in health policy research. While existing qualitative research manuals offer direction for conducting document analysis, there has been little specific discussion about how to use this method to understand and analyse health policy. Drawing on guidance from other disciplines and our own research experience, we present a systematic approach for document analysis in health policy research called the READ approach: (1) ready your materials, (2) extract data, (3) analyse data and (4) distil your findings. We provide practical advice on each step, with consideration of epistemological and theoretical issues such as the socially constructed nature of documents and their role in modern bureaucracies. We provide examples of document analysis from two case studies from our work in Pakistan and Niger in which documents provided critical insight and advanced empirical and theoretical understanding of a health policy issue. Coding tools for each case study are included as Supplementary Files to inspire and guide future research. These case studies illustrate the value of rigorous document analysis to understand policy content and processes and discourse around policy, in ways that are either not possible using other methods, or greatly enrich other methods such as in-depth interviews and observation. Given the central nature of documents to health policy research and importance of reading them critically, the READ approach provides practical guidance on gaining the most out of documents and ensuring rigour in document analysis.

**Key Messages**

- Rigour in qualitative research is judged partly by the use of deliberate, systematic procedures; however, little specific guidance is available for analysing documents, a nonetheless common method in health policy research.
- Document analysis is useful for understanding policy content across time and geographies, documenting processes, triangulating with interviews and other sources of data, understanding how information and ideas are presented formally, and understanding issue framing, among other purposes.
- The READ (Ready materials, Extract data, Analyse data, Distil) approach provides a step-by-step guide to conducting document analysis for qualitative policy research.
- The READ approach can be adapted to different purposes and types of research, two examples of which are presented in this article, with sample tools in the Supplementary Materials.

## Introduction

Document analysis (also called document review) is one of the most commonly used methods in health policy research; it is nearly impossible to conduct policy research without it. Writing in early 20th century, Weber (2015) identified the importance of formal, written documents as a key characteristic of the bureaucracies by which modern societies function, including in public health. Accordingly, critical social research has a long tradition of documentary review: Marx analysed official reports, laws, statues, census reports and newspapers and periodicals over a nearly 50-year period to come to his world-altering conclusions (Harvey, 1990). Yet in much of social science research, ‘documents are placed at the margins of consideration,’ with privilege given to the spoken word via methods such as interviews, possibly due to the fact that many qualitative methods were developed in the anthropological tradition to study mainly pre-literate societies (Prior, 2003). To date, little specific guidance is available to help health policy researchers make the most of these wells of information. 

The term ‘documents’ is defined here broadly, following Prior, as physical or virtual artefacts designed by creators, for users, to function within a particular setting (Prior, 2003). Documents exist not as standalone objects of study but must be understood in the social web of meaning within which they are produced and consumed. For example, some analysts distinguish between public documents (produced in the context of public sector activities), private documents (from business and civil society) and personal documents (created by or for individuals, and generally not meant for public consumption) (Mogalakwe, 2009). Documents can be used in a number of ways throughout the research process (Bowen, 2009). In the planning or study design phase, they can be used to gather background information and help refine the research question. Documents can also be used to spark ideas for disseminating research once it is complete, by observing the ways those who will use the research speak to and communicate ideas with one another.

Documents can also be used during data collection and analysis to help answer research questions. Recent health policy research shows that this can be done in at least four ways. Frequently, policy documents are reviewed to describe the content or categorize the approaches to specific health problems in existing policies, as in reviews of the composition of drowning prevention resources in the United States or policy responses to foetal alcohol spectrum disorder in South Africa (Katchmarchi *et al.*, 2018; Adebiyi *et al.*, 2019). In other cases, non-policy documents are used to examine the implementation of health policies in real-world settings, as in a review of web sources and newspapers analysing the functioning of community health councils in New Zealand (Gurung *et al.*, 2020). Perhaps less frequently, document analysis is used to analyse policy processes, as in an assessment of multi-sectoral planning process for nutrition in Burkina Faso (Ouedraogo *et al.*, 2020). Finally, and most broadly, document analysis can be used to inform new policies, as in one study that assessed cigarette sticks as communication and branding ‘documents,’ to suggest avenues for further regulation and tobacco control activities (Smith *et al.*, 2017).

This practice paper provides an overarching method for conducting document analysis, which can be adapted to a multitude of research questions and topics. Document analysis is used in most or all policy studies; the aim of this article is to provide a systematized method that will enhance procedural rigour. We provide an overview of document analysis, drawing on guidance from disciplines adjacent to public health, introduce the ‘READ’ approach to document analysis and provide two short case studies demonstrating how document analysis can be applied.

## What is document analysis?

Document analysis is a systematic procedure for reviewing or evaluating documents, which can be used to provide context, generate questions, supplement other types of research data, track change over time and corroborate other sources (Bowen, 2009). In one commonly cited approach in social research, Bowen recommends first skimming the documents to get an overview, then reading to identify relevant categories of analysis for the overall set of documents and finally interpreting the body of documents (Bowen, 2009). Document analysis can include both quantitative and qualitative components: the approach presented here can be used with either set of methods, but we emphasize qualitative ones, which are more adapted to the socially constructed meaning-making inherent to collaborative exercises such as policymaking.

The study of documents as a research method is common to a number of social science disciplines—yet in many of these fields, including sociology (Mogalakwe, 2009), anthropology (Prior, 2003) and political science (Wesley, 2010), document-based research is described as ill-considered and underutilized. Unsurprisingly, textual analysis is perhaps most developed in fields such as media studies, cultural studies and literary theory, all disciplines that recognize documents as ‘social facts’ that are created, consumed, shared and utilized in socially organized ways (Atkinson and Coffey, 1997). Documents exist within social ‘fields of action,’ a term used to designate the environments within which individuals and groups interact. Documents are therefore not mere records of social life, but integral parts of it—and indeed can become agents in their own right (Prior, 2003). Powerful entities also manipulate the nature and content of knowledge; therefore, gaps in available information must be understood as reflecting and potentially reinforcing societal power relations (Bryman and Burgess, 1994).

Document analysis, like any research method, can be subject to concerns regarding validity, reliability, authenticity, motivated authorship, lack of representativity and so on. However, these can be mitigated or avoided using standard techniques to enhance qualitative rigour, such as triangulation (within documents and across methods and theoretical perspectives), ensuring adequate sample size or ‘engagement’ with the documents, member checking, peer debriefing and so on (Maxwell, 2005).

Document analysis can be used as a standalone method, e.g. to analyse the contents of specific types of policy as they evolve over time and differ across geographies, but document analysis can also be powerfully combined with other types of methods to cross-validate (i.e. triangulate) and deepen the value of concurrent methods. As one guide to public policy research puts it, ‘almost all likely sources of information, data, and ideas fall into two general types: documents and people’ (Bardach and Patashnik, 2015). Thus, researchers can ask interviewees to address questions that arise from policy documents and point the way to useful new documents. Bardach and Patashnik suggest alternating between documents and interviews as sources as information, as one tends to lead to the other, such as by scanning interviewees’ bookshelves and papers for titles and author names (Bardach and Patashnik, 2015). Depending on your research questions, document analysis can be used in combination with different types of interviews (Berner-Rodoreda *et al.*, 2018), observation (Harvey, 2018), and quantitative analyses, among other common methods in policy research.

## The READ approach

The READ approach to document analysis is a systematic procedure for collecting documents and gaining information from them in the context of health policy studies at any level (global, national, local, etc.). The steps consist of: (1) ready your materials, (2) extract data, (3) analyse data and (4) distil your findings. We describe each of these steps in turn.

### Step 1. Ready your materials

At the outset, researchers must set parameters in terms of the nature and number (approximately) of documents they plan to analyse, based on the research question. How much time will you allocate to the document analysis, and what is the scope of your research question? Depending on the answers to these questions, criteria should be established around (1) the topic (a particular policy, programme, or health issue, narrowly defined according to the research question); (2) dates of inclusion (whether taking the long view of several decades, or zooming in on a specific event or period in time); and (3) an indicative list of places to search for documents (possibilities include databases such as Ministry archives; LexisNexis or other databases; online searches; and particularly interview subjects). For difficult-to-obtain working documents or otherwise non-public items, bringing a flash drive to interviews is one of the best ways to gain access to valuable documents.

For research focusing on a single policy or programme, you may review only a handful of documents. However, if you are looking at multiple policies, health issues, or contexts, or reviewing shorter documents (such as newspaper articles), you may look at hundreds, or even thousands of documents. When considering the number of documents you will analyse, you should make notes on the type of information you plan to extract from documents—i.e. what it is you hope to learn, and how this will help answer your research question(s). The initial criteria—and the data you seek to extract from documents—will likely evolve over the course of the research, as it becomes clear whether they will yield too few documents and information (a rare outcome), far too many documents and too much information (a much more common outcome) or documents that fail to address the research question; however, it is important to have a starting point to guide the search. If you find that the documents you need are unavailable, you may need to reassess your research questions or consider other methods of inquiry. If you have too many documents, you can either analyse a subset of these (Panel 1) or adopt more stringent inclusion criteria.

**Panel 1:** Exploring the framing of diseases in Pakistani media

Health policies must account for how societies perceive and understand a given disease’s origins and causes, and media sources play an important role in framing health issues (Nelkin, 1991; Entman, 1993). Document analysis was employed to understand the frames used in print media (newspapers) in Pakistan when discussing Human Immunodeficiency Virus (HIV) and viral hepatitis, two diseases that are spread using similar modes of transmission but have varying levels of stigma in the country. Alongside document analysis, key informant interviews were used for triangulation and to flesh out what stigma for HIV meant in the country. A sample of newspaper articles was drawn from the electronic database LexisNexis (January 2006-September 2016) based on readership, electronic availability in LexisNexis and geographic diversity, to capture cultural differences across provinces over time (Strömbäck and Dimitrova, 2011). Broad search terms were used for HIV and viral hepatitis, resulting in 3415 articles for hepatitis and1580 articles for HIV. A random sample comprising 10% of the total HIV articles (*n* = 156) and 5% of the total hepatitis articles (*n* = 176) was selected and coded using a fixed coding guide. The coding guide was developed using an inductive approach (Krippendorff, 2004; Mayring, 2004), which involved reading a sample of articles line by line to identify media frames for HIV and viral hepatitis (Abdelmutti and Hoffman-Goetz, 2009; Claassen *et al.*, 2012). Two rounds of pre-testing were carried out before the final sample of articles was coded. However, the use of LexisNexis as the primary data source excluded newspapers published in the local language (opening up the possibility of omitting some media frames). Therefore, interviews were important for triangulation of findings. Data from document analysis were collated in an Excel sheet and analysed in STATA 14. The findings of the document analysis highlighted that while both diseases were transmitted predominantly through injecting drug use in the country, hepatitis was only discussed using frames such as ‘medical’ (discussing transmission, prevention, and treatment methods), ‘resources’ (resources available to fight the disease), ‘magnitude’ (gives the scope of the problem or disease prevalence) and ‘need for awareness’–there was no ‘stigma and discrimination’ frame attached to the disease [Figure, HIV and viral hepatitis articles by main frames (%)]. In contrast, the ‘stigma and discrimination’ frame and the ‘social causes of disease’ frame (discussing non-medical causes for the spread of disease) were used exclusively in articles on HIV, notably including suggestions that acquiring the disease was linked to socially immoral and un-Islamic behaviour. Key informant interviews helped to probe further the traits associated with someone who had HIV. Taken together, document analysis and key informant interviews helped build a richer narrative of HIV stigma in the country. Given the difference in how these diseases were understood, these findings suggested that there was a need for explicit policy to reframe HIV as a disease. Countries such as Iran, Indonesia and Malaysia have successfully garnered government and policy attention to HIV and reduced stigma by reframing it as a disease spread through injecting drug use (Kamarulzaman, 2013).

In Table 1, we present a non-exhaustive list of the types of documents that can be included in document analyses of health policy issues. In most cases, this will mean written sources (policies, reports, articles). The types of documents to be analysed will vary by study and according to the research question, although in many cases, it will be useful to consult a mix of formal documents (such as official policies, laws or strategies), ‘gray literature’ (organizational materials such as reports, evaluations and white papers produced outside formal publication channels) and, whenever possible, informal or working documents (such as meeting notes, PowerPoint presentations and memoranda). These latter in particular can provide rich veins of insight into how policy actors are thinking through the issues under study, particularly for the lucky researcher who obtains working documents with ‘Track Changes.’ How you prioritize documents will depend on your research question: you may prioritize official policy documents if you are studying policy content, or you may prioritize informal documents if you are studying policy process.

**Table 1:** Types of documents that can be consulted in studies of health policy

Category	Examples
Official documents	Policies or policy directives Strategies for sectors or on specific health problems Official statements and declarations Official position papers Statistical surveys or publications
Implementation documents	Training manuals or work tools (booklets, clinical files, etc.) Midterm/final reports or evaluations Financial analyses Operational plans Project proposals Funding requests
Legal documents	Laws Regulations Memorandums of understanding Cooperation agreements
Working documents	Meeting reports or minutes Memoranda Committee reports PowerPoint presentations Draft documents Mission reports Emails
Scholarly work	Scientific or peer-reviewed publications Masters or doctoral dissertations Textbooks and other course materials
Media and communications	Newspaper and magazine articles Podcasts, videos and radio and television segments Advertisements and posters Newsletters, bulletins, listservs, blogs and webpages Twitter conversations and other social media
Other	Promotional materials (pens, notebooks, lanyards, etc.) Warning labels and nutritional labels on food and other products Medical or other health devices Floor plans, architectural plans and maps

During this initial preparatory phase, we also recommend devising a file-naming system for your documents (e.g. Author.Date.Topic.Institution.PDF), so that documents can be easily retrieved throughout the research process. After extracting data and processing your documents the first time around, you will likely have additional ‘questions’ to ask your documents and need to consult them again. For this reason, it is important to clearly name source files and link filenames to the data that you are extracting (see sample naming conventions in the Supplementary Materials).

### Step 2. Extract data

Data can be extracted in a number of ways, and the method you select for doing so will depend on your research question and the nature of your documents. One simple way is to use an Excel spreadsheet where each row is a document and each column is a category of information you are seeking to extract, from more basic data such as the document title, author and date, to theoretical or conceptual categories deriving from your research question, operating theory or analytical framework (Panel 2). Documents can also be imported into thematic coding software such as Atlas.ti or NVivo, and data extracted that way. Alternatively, if the research question focuses on process, documents can be used to compile a timeline of events, to trace processes across time. Ask yourself, how can I organize these data in the most coherent manner? What are my priority categories? We have included two different examples of data extraction tools in the Supplementary Materials to this article to spark ideas.

**Panel 2:** Case study Documents tell part of the story in Niger

In a multi-country policy analysis of integrated Community Case Management of childhood illness (iCCM), Niger was among the few countries that scaled up the policy at national level (Bennett et al., 2015). Alongside key stakeholder interviews and non-participant observation, document analysis was used to reconstruct the policy process leading to this outcome. In total, 103 documents were obtained from policy actors in Niger, researchers working on similar topics, or collected on the Internet (Dalglish et al., 2015). Documents included official policies and strategies, field reports, legal regulations, program evaluations, funding proposals, newsletters and newspaper articles, among other sources. Document acquisition was greatly facilitated by asking for documents during stakeholder interviews, although some documents were not available due to a fire that destroyed World Health Organization (WHO) servers in the years preceding the study. Data from the documents was extracted into a Microsoft Excel file, recording information about specific aspects of child health policy and programs, framing of issues, use of research evidence, and mention of international recommendations, among other topics. Documents were also used to compile a timeline of events in the policy process. Policy processes were elucidated by creating a timeline of events, which documented how specific decrees, workshops, meetings, and other events occurred over time. The timeline was overlaid with measures of implementation (number of health posts built, number of health workers trained) to understand how decision-making processes propelled real-world outcomes, and served as proxies for financial data that were rarely included in policy documents (Dalglish et al., 2015). Additionally, document analysis revealed a partial account of what was driving these events. Many documents showed a concern for reaching the Millennium Development Goal on child mortality (Figure, Representations of progress toward Millennium Development Goal 4 in Nigerien policy documents). Graphs mapping country progress toward Millennium Development Goal (MDG)-4 appeared in nearly all documentation on iCCM, and progress was regularly reported on by the Nigerien National Institute of Statistics, suggesting that these were a significant motivating factor in policy and resource allocation decisions. Yet older historical documents showed a long-standing recognition of the problem of children's access to life-saving healthcare (well before the MDGs), with policy remedies going back to least 1965 in the form of rural first-aid workers (Fournier and Djermakoye, 1975). Triangulation with interviews and observation also showed that national policymakers’ practical knowledge and ethical imperative to save children's lives was at least as important as the MDGs in motivating policy action (Dalglish et al., 2017). Taken together, the document and non-document data showed that, as in other contexts, the MDGs were useful mainly to direct international fundraising and satisfy donor norms in expectation of funding increases (Marten, 2019).

Document analyses are first and foremost exercises in close reading: documents should be read thoroughly, from start to finish, including annexes, which may seem tedious but which sometimes produce golden nuggets of information. Read for overall meaning as you extract specific data related to your research question. As you go along, you will begin to have ideas or build working theories about what you are learning and observing in the data. We suggest capturing these emerging theories in extended notes or ‘memos,’ as used in Grounded Theory methodology (Charmaz, 2006); these can be useful analytical units in themselves and can also provide a basis for later report and article writing.

As you read more documents, you may find that your data extraction tool needs to be modified to capture all the relevant information (or to avoid wasting time capturing irrelevant information). This may require you to go back and seek information in documents you have already read and processed, which will be greatly facilitated by a coherent file-naming system. It is also useful to keep notes on other documents that are mentioned that should be tracked down (sometimes you can write the author for help). As a general rule, we suggest being parsimonious when selecting initial categories to extract from data. Simply reading the documents takes significant time in and of itself—make sure you think about how, exactly, the specific data you are extracting will be used and how it goes towards answering your research questions.

### Step 3. Analyse data

As in all types of qualitative research, data collection and analysis are iterative and characterized by emergent design, meaning that developing findings continually inform whether and how to obtain and interpret data (Creswell, 2013). In practice, this means that during the data extraction phase, the researcher is already analysing data and forming initial theories—as well as potentially modifying document selection criteria. However, only when data extraction is complete can one see the full picture. For example, are there any documents that you would have expected to find, but did not? Why do you think they might be missing? Are there temporal trends (i.e. similarities, differences or evolutions that stand out when documents are ordered chronologically)? What else do you notice? We provide a list of overarching questions you should think about when viewing your body of document as a whole (Table 2). 

**Table 2:** Questions to ask your overall body of documents

*When analysing individual documents*: Is the document complete? Is this a finished document, or a draft? What is the purpose of the document? Who is its target audience? Under which circumstances was the document produced? Under which circumstances is it consumed? Who created the document? Aside from the listed authors, what other contributors were likely involved in its creation? What could be the ‘agenda’ of the document’s creators? Are there other versions of the document? Why? How might they differ? Are there internal contradictions within the document (e.g. differing rationales or framings)? Is the document credible? Do you have any questions about its accuracy, good faith, balance, selective reasoning, etc.? What sources are cited (or not cited)? What kind of evidence does it use?
*When analysing the overall body of documents:* How complete is the set of documents? What is missing? Which documents were easy to find? Which were harder to find? Which proved impossible to find? Why might this be the case? Which voices are represented in the overall body of documents? Which are not? How do the documents compare in terms of content? How do they compare in terms of style, format, length and ‘look’? How about in terms of formality and tone? What visual information can you find in the documents (charts and graphs, pictures, etc.)? How are the same issues discussed in different ways across documents? Do the documents ‘speak to each other’? Do they reference each other, or respond to each other’s arguments or propositions? Are they responding to other documents not included in the review? How are documents similar or different across different topic areas, types of document or governance levels (e.g. global, national, sub-national)? How does the information from documents compare to data from other data sources (e.g. interviews, focus groups, observation, quantitative analyses)?

**Figure:**
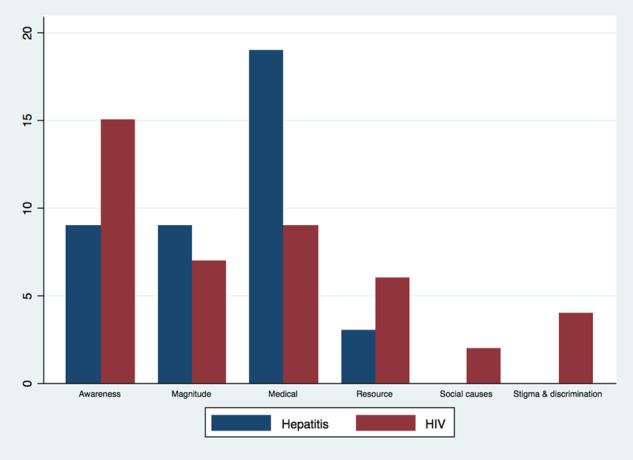
HIV and viral hepatitis articles by main frames (%). Note: The percentage of articles is calculated by dividing the number of articles appearing in each frame for viral hepatitis and HIV by the respectivenumber of sampled articles for each disease (N = 137 for HIV; N = 117 for hepatitis). Time frame: 1 January 2006 to 30 September 2016

**Figure:**
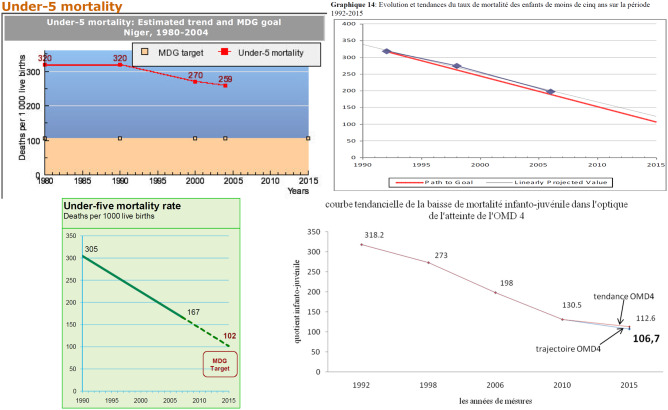
Representations of progress toward Millennium Development Goal 4 in Nigerien policy documents. Sources: clockwise from upper left: (WHO 2006); (Institut National de la Statistique 2010); (Ministè re de la Santé Publique 2010); (Unicef 2010)

In addition to the meaning-making processes you are already engaged in during the data extraction process, in most cases, it will be useful to apply specific analysis methodologies to the overall corpus of your documents, such as policy analysis (Buse *et al.*, 2005). An array of analysis methodologies can be used, both quantitative and qualitative, including case study methodology, thematic content analysis, discourse analysis, framework analysis and process tracing, which may require differing levels of familiarity and skills to apply (we highlight a few of these in the case studies below). Analysis can also be structured according to theoretical approaches. When it comes to analysing policies, process tracing can be particularly useful to combine multiple sources of information, establish a chronicle of events and reveal political and social processes, so as to create a narrative of the policy cycle (Yin, 1994; Shiffman *et al.*, 2004). Practically, you will also want to take a holistic view of the documents’ ‘answers’ to the questions or analysis categories you applied during the data extraction phase. Overall, what did the documents ‘say’ about these thematic categories? What variation did you find within and between documents, and along which axes? Answers to these questions are best recorded by developing notes or memos, which again will come in handy as you write up your results.

As with all qualitative research, you will want to consider your own positionality towards the documents (and their sources and authors); it may be helpful to keep a ‘reflexivity’ memo documenting how your personal characteristics or pre-standing views might influence your analysis (Watt, 2007).

### Step 4. Distil your findings

You will know when you have completed your document review when one of the three things happens: (1) completeness (you feel satisfied you have obtained every document fitting your criteria—this is rare), (2) out of time (this means you should have used more specific criteria), and (3) saturation (you fully or sufficiently understand the phenomenon you are studying). In all cases, you should strive to make the third situation the reason for ending your document review, though this will not always mean you will have read and analysed every document fitting your criteria—just enough documents to feel confident you have found good answers to your research questions.

Now it is time to refine your findings. During the extraction phase, you did the equivalent of walking along the beach, noticing the beautiful shells, driftwood and sea glass, and picking them up along the way. During the analysis phase, you started sorting these items into different buckets (your analysis categories) and building increasingly detailed collections. Now you have returned home from the beach, and it is time to clean your objects, rinse them of sand and preserve only the best specimens for presentation. To do this, you can return to your memos, refine them, illustrate them with graphics and quotes and fill in any incomplete areas. It can also be illuminating to look across different strands of work: e.g. how did the content, style, authorship, or tone of arguments evolve over time? Can you illustrate which words, concepts or phrases were used by authors or author groups?

Results will often first be grouped by theoretical or analytic category, or presented as a policy narrative, interweaving strands from other methods you may have used (interviews, observation, etc.). It can also be helpful to create conceptual charts and graphs, especially as this corresponds to your analytical framework (Panels 1 and 2). If you have been keeping a timeline of events, you can seek out any missing information from other sources. Finally, ask yourself how the validity of your findings checks against what you have learned using other methods. The final products of the distillation process will vary by research study, but they will invariably allow you to state your findings relative to your research questions and to draw policy-relevant conclusions.

## Conclusion

Document analysis is an essential component of health policy research—it is also relatively convenient and can be low cost. Using an organized system of analysis enhances the document analysis’s procedural rigour, allows for a fuller understanding of policy process and content and enhances the effectiveness of other methods such as interviews and non-participant observation. We propose the READ approach as a systematic method for interrogating documents and extracting study-relevant data that is flexible enough to accommodate many types of research questions. We hope that this article encourages discussion about how to make best use of data from documents when researching health policy questions.

## Supplementary Data

Supplementary data are available at *Health Policy and Planning* online.

## Supplementary Material

czaa064_supplementary_data

## References

[czaa064-B1] Abdelmutti N , Hoffman-Goetz L. 2009 Risk messages about HPV, cervical cancer, and the HPV vaccine Gardasil: a content analysis of Canadian and U.S. national newspaper articles. Women & Health 49: 422–40. 19851946 10.1080/03630240903238776

[czaa064-B2] Adebiyi BO , Mukumbang FC, Beytell A-M. 2019 To what extent is fetal alcohol spectrum disorder considered in policy-related documents in South Africa? A document review. Health Research Policy and Systems 17:10.1186/s12961-019-0447-9 PMC6489263 31036004

[czaa064-B3] Atkinson PA , Coffey A. 1997 Analysing documentary realities In: Silverman D (ed). Qualitative Research: Theory, Method and Practice. London: SAGE.

[czaa064-B4] Bardach E , Patashnik EM. 2015 Practical Guide for Policy Analysis: The Eightfold Path to More Effective Problem Solving. Los Angeles: SAGE.

[czaa064-B5] Bennett S , Dalglish SL, Juma PA, Rodríguez DC. 2015 Altogether now… understanding the role of international organizations in iCCM policy transfer. Health Policy and Planning 30: ii26–35. 26516147 10.1093/heapol/czv071

[czaa064-B6] Berner-Rodoreda A , Bärnighausen T, Kennedy C et al 2018 From doxastic to epistemic: a typology and critique of qualitative interview styles. Qualitative Inquiry 26: 291–305. 1077800418810724. 32038093 10.1177/1077800418810724 PMC6985996

[czaa064-B7] Bowen GA. 2009 Document analysis as a qualitative research method. Qualitative Research Journal 9: 27–40.

[czaa064-B8] Bryman A. 1994 *Analyzing Qualitative Data*.

[czaa064-B9] Buse K , Mays N, Walt G. 2005 Making Health Policy. New York: Open University Press.

[czaa064-B10] Charmaz K. 2006 Constructing Grounded Theory: A Practical Guide through Qualitative Analysis. London: SAGE.

[czaa064-B11] Claassen L , Smid T, Woudenberg F, Timmermans DRM. 2012 Media coverage on electromagnetic fields and health: content analysis of Dutch newspaper articles and websites. Health, Risk & Society 14: 681–96.

[czaa064-B12] Creswell JW. 2013 Qualitative Inquiry and Research Design. Thousand Oaks, CA: SAGE.

[czaa064-B13] Dalglish SL , Rodríguez DC, Harouna A, Surkan PJ. 2017 Knowledge and power in policy-making for child survival in Niger. Social Science & Medicine 177: 150–7. 28167340 10.1016/j.socscimed.2017.01.056

[czaa064-B14] Dalglish SL , Surkan PJ, Diarra A, Harouna A, Bennett S. 2015 Power and pro-poor policies: the case of iCCM in Niger. Health Policy and Planning 30: ii84–94. 26516154 10.1093/heapol/czv064

[czaa064-B15] Entman RM. 1993 Framing: toward clarification of a fractured paradigm. Journal of Communication 43: 51–8.

[czaa064-B16] Fournier G , Djermakoye IA. 1975 Village health teams in Niger (Maradi Department) In: Newell KW (ed). Health by the People. Geneva: WHO.

[czaa064-B17] Gurung G , Derrett S, Gauld R. 2020 The role and functions of community health councils in New Zealand’s health system: a document analysis. The New Zealand Medical Journal 133: 70–82. 32078603

[czaa064-B18] Harvey L. 1990 Critical Social Research. London: Unwin Hyman.

[czaa064-B19] Harvey SA. 2018 Observe before you leap: why observation provides critical insights for formative research and intervention design that you’ll never get from focus groups, interviews, or KAP surveys. Global Health: Science and Practice 6: 299–316. 10.9745/GHSP-D-17-00328 PMC6024634 29794000

[czaa064-B118] Institut National de la Statistique. 2010. Rapport National sur les Progrès vers l'atteinte des Objectifs du Millénaire pour le Développement. Niamey, Niger: INS.

[czaa064-B20] Kamarulzaman A. 2013 Fighting the HIV epidemic in the Islamic world. Lancet 381: 2058–60. 23769216 10.1016/S0140-6736(13)61033-8

[czaa064-B21] Katchmarchi AB , Taliaferro AR, Kipfer HJ. 2018 A document analysis of drowning prevention education resources in the United States. International Journal of Injury Control and Safety Promotion 25: 78–84. 28701104 10.1080/17457300.2017.1341932

[czaa064-B22] Krippendorff K. 2004 Content Analysis: An Introduction to Its Methodology. SAGE.

[czaa064-B23] Marten R. 2019 How states exerted power to create the Millennium Development Goals and how this shaped the global health agenda: lessons for the sustainable development goals and the future of global health. Global Public Health 14: 584–99. 29697307 10.1080/17441692.2018.1468474

[czaa064-B24] Maxwell JA. 2005 Qualitative Research Design: An Interactive Approach, 2nd edn. Thousand Oaks, CA: Sage Publications.

[czaa064-B25] Mayring P. 2004 Qualitative Content Analysis. In: Flick U, von Kardorff E, Steinke I (eds). *A Companion to Qualitative Research* SAGE.

[czaa064-B126] Ministère de la Santé Publique. 2010. Enquête nationale sur la survie des enfants de 0 à 59 mois et la mortalité au Niger 2010. Niamey, Niger: MSP.

[czaa064-B26] Mogalakwe M. 2009 The documentary research method—using documentary sources in social research. Eastern Africa Social Science Research Review 25: 43–58.

[czaa064-B27] Nelkin D. 1991 AIDS and the news media. The Milbank Quarterly 69: 293–307. 1791792

[czaa064-B28] Ouedraogo O , Doudou MH, Drabo KM et al 2020 Policy overview of the multisectoral nutrition planning process: the progress, challenges, and lessons learned from Burkina Faso. The International Journal of Health Planning and Management 35: 120–39. 31271224 10.1002/hpm.2823

[czaa064-B29] Prior L. 2003 Using Documents in Social Research. London: SAGE.

[czaa064-B30] Shiffman J , Stanton C, Salazar AP. 2004 The emergence of political priority for safe motherhood in Honduras. Health Policy and Planning 19: 380–90. 15459163 10.1093/heapol/czh053

[czaa064-B31] Smith KC , Washington C, Welding K et al 2017 Cigarette stick as valuable communicative real estate: a content analysis of cigarettes from 14 low-income and middle-income countries. Tobacco Control 26: 604–7. 10.1136/tobaccocontrol-2016-053148 PMC5574399 27534777

[czaa064-B32] Strömbäck J , Dimitrova DV. 2011 Mediatization and media interventionism: a comparative analysis of Sweden and the United States. The International Journal of Press/Politics 16: 30–49.

[czaa064-B33] Watt D. 2007 On becoming a qualitative researcher: the value of reflexivity. Qualitative Report 12: 82–101.

[czaa064-B34] Weber M. 2015 Bureaucracy In: Waters T, Waters D (eds). Rationalism and Modern Society: New Translations on Politics, Bureaucracy, and Social Stratification. London: Palgrave MacMillan.

[czaa064-B35] Wesley JJ. 2010. Qualitative Document Analysis in Political Science.

[czaa064-B135] World Health Organization. 2006. Country Health System Fact Sheet 2006: Niger. Niamey, Niger: WHO.

[czaa064-B36] Yin R. 1994 Case Study Research: Design and Methods. Thousand Oaks, CA: Sage.

