# Supplementary material for: Document analysis in health policy research: the READ approach
Source: Health Policy Plan. 2020 Nov 11;35(10):1424–31. doi: 10.1093/heapol/czaa064 (PMC7886435; doi:10.1093/heapol/czaa064)
Supplement: czaa064_supplementary_data [file czaa064_supplementary_data.zip › #Supplementary materials 0 (File naming).docx]

File naming conventions

Start a folder on your computer for your documents and decide on your naming system. There are many ways to do this, the important thing is to choose one system and be consistent. For example, you could choose to name your files like this:

- Author_Year_Subject_Institution

🡪 Ibrahim_2016_Maternal child health policy_MOH.PDF

Or like this:

- Row in Document analysis spreadsheet. Institution.Year

🡪 34.WorldBank.2020.DOCX

This might make your computer folder look something like this:


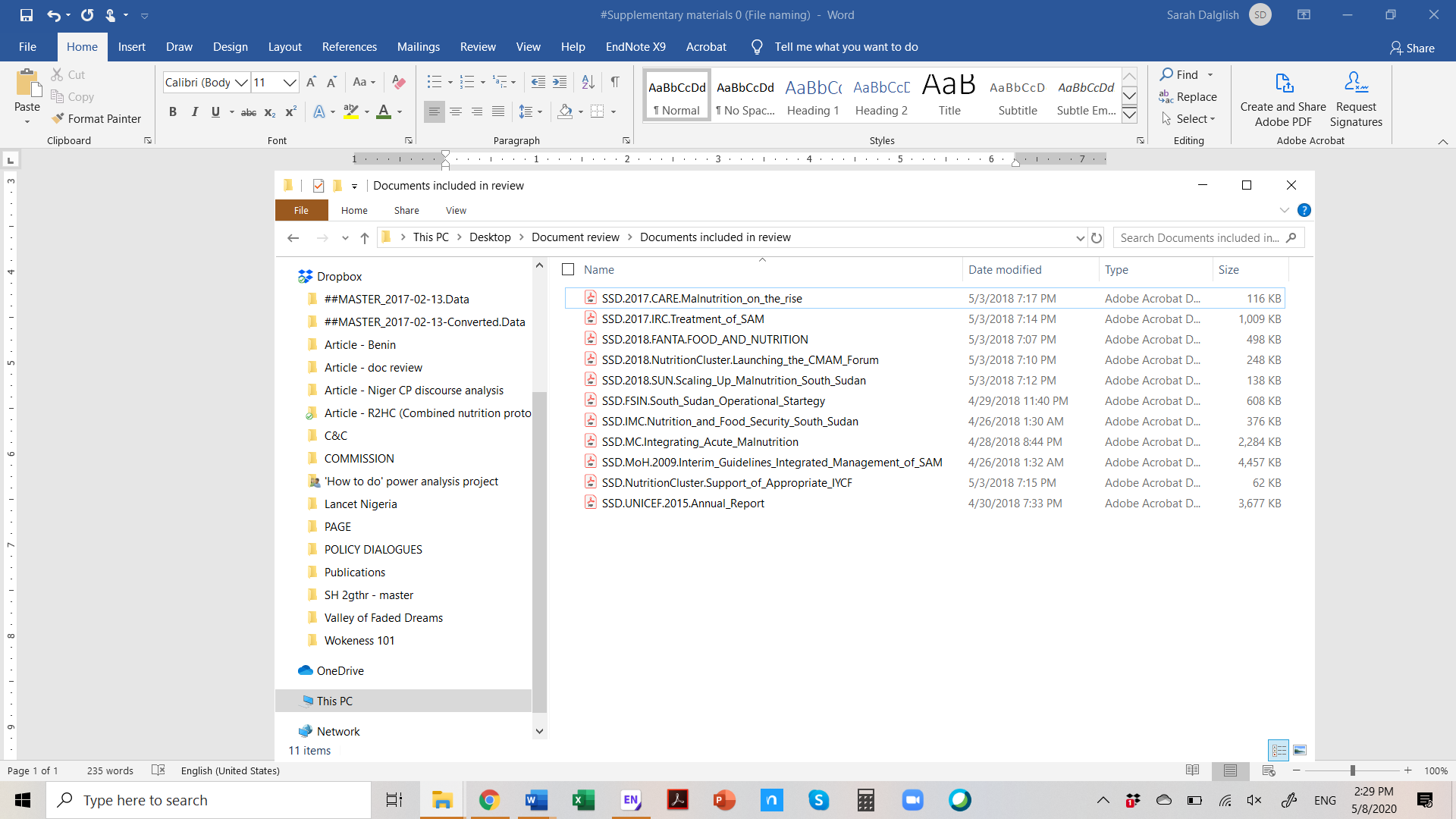


While such naming conventions can make it easy to find files, they can also sometimes lead to long filenames. Another option is to apply shortened codes. For example:

- Each filename will consist of three parts: a country code (3 letters), a date (4 numbers), a publication type code (3 letters) and a document number (2 numbers):
  - *Country code* = code for country of origin (e.g., FRA = France, SEN = Senegal, etc.)
  - *Date (year)* = 2011, 2020, etc.
  - *Document type code* = indicates which type of document it is (e.g. NEW for a news item, POL for an official policy)
  - *Doc number* = the number of that type of document that you’ve collected
- Examples of what this would look like:
  - FRA2017NEW13.pdf
  - SEN2020POL03.pdf

Spend some time playing around with different options to see what will work best for your study – it is time well spent. **Remember your goal:** **to be able to find things easily later!**
