# Supplementary material for: Document analysis in health policy research: the READ approach
Source: Health Policy Plan. 2020 Nov 11;35(10):1424–31. doi: 10.1093/heapol/czaa064 (PMC7886435; doi:10.1093/heapol/czaa064)
Supplement: czaa064_supplementary_data [file czaa064_supplementary_data.zip › #Supplementary materials 1 (Pakistan).docx]

**PROCEDURE FOR DEVELOPING THE CODING GUIDE:**

Random samples of newspaper articles were taken from the population of both HIV and hepatitis articles, and were read to determine the frames used for discussing the diseases. Following the initial read of the articles, frames were developed for categorizing the thematic focus of newspaper stories. Generating frames was an iterative process and once the frames were generated, the coding guide was pre-tested on a subset of articles to determine if the decision rules meaningfully categorized the articles. Two rounds of pre-testing were carried out following initial development of the coding guide. In the first round of pre-testing, several new decision rules for classifying the frames emerged while in the second round no new frames or decision rules emerged.

An identical coding guide was used for coding hepatitis. The only difference across the coding guides was replacing the disease name.

**CODING GUIDE FOR CONTENT ANALYSIS**

**Article ID**

[*ID*] List the article’s unique identifier

[YYYYMMDD] [Newspaper name] [Headline]

**PROVINCE / SCOPE**

1. Punjab
2. Sindh
3. Balochistan
4. Khyber Pukhtoonkhawa (KPK)
5. Global

- *I define something as the* ***main frame*** *of the article if it is in the headline and it is the dominant topic of discussion in the article.*
- ***Peripheral frame*** *is when the dominant discussion is not on HIV OR the main frame has HIV (for example: medical or resource etc.) but a small discussion is also on some other frame ( for example stigma). An article can have multiple peripheral frames. A peripheral frame can vary in length from a mention of HIV in one sentence (but of relevance) to an entire paragraph written on HIV.*
- ***Not relevant*** *for a main frame and peripheral frame is when there is no substantive mention of HIV. For example, “ In connection with TB, the manager of HIV department said x*”
- ***Other*** *is not captured in the existing frames but is a discussion on HIV/HIV using another frame.*

**MAIN FRAME**

1. Medical frame
2. Resource frame
3. Awareness frame
4. Magnitude frame
5. Stigma and discrimination frame
6. Social causes of HIV frame
7. HIV not in main frame
8. Not relevant
9. Other _______________

**PREIPHERAL FRAME**

1. Medical frame
2. Resource frame
3. Awareness frame
4. Magnitude frame
5. Stigma and discrimination frame
6. Social causes of HIV frame
7. Not relevant
8. Other ______________

**MEDICAL FRAME**

*I define a frame as medical when there is discussion on treatment, transmission methods, prevention methods. This can be a discussion on exiting treatment, prevention or the need for treatment and prevention. This discussion can be by a doctor, policy maker or someone simply giving a general report on any of the aforementioned topics.*

A frame is medical if it does one **or** more of the following:

- It talks about HIV treatment (for example: ARV, medicines etc.)
- It talks about the need for treatment (for example: ARV, medicines etc.)
- It talks about diagnoses
- It talks about modes of transmission (for example: sex, injecting drug use, blood and blood products, sharing needles, etc.)
- It talks about modes of prevention (for example: avoiding sex, drug use, blood and blood products, sharing needles)
- It talks about HIV risk groups: sex workers, injecting drug users, deportees, prisoners etc.

**RESOURCE FRAME**

*I define a frame as a resource frame when there is discussion on resources, resource utilization or lack of resources to address the disease. These resources may be human, financial, technical or institutional.*

A frame is resource if it does one **or** more of the following:

- It talks about resource gaps (shortage of medicines, treatment, infrastructure)
- It talks about patients registered in hospitals
- It talks about patients not registered in hospitals
- It talks about budget/expenditure on HIV
- It talks about existing support/need for support from the local and international community

**AWARENESS FRAME**

*An awareness frame refers to a frame that talks about existing awareness efforts, the need for awareness efforts for HIV.*

A frame is awareness if it does one **or** more of the following:

- It talks about awareness efforts (for example: education campaigns, adds, press-conferences, walks, seminars and workshops to increase awareness)
- It talks about the need for awareness efforts or the lack of awareness

**MAGNITUDE FRAME**

*This refers to a frame that gives a sense of the scope of the HIV problem at hand. It does this quantitatively, or qualitatively.*

A frame is magnitude if it does one **or** more of the following

- It gives a statistic on HIV prevalence
- It gives a statistic on HIV infections
- It gives a statistic on patients that are registered / need to be registered at the hospital
- It gives a statistic on the number of HIV risk groups
- It talks about HIV as a pressing issue
- It gives a sense of HIV being a pressing issue qualitatively by using words such as deadly, killer, dead, critical, alarming, fatal

**STIGMA AND DISCRIMINATION FRAME**

*This frame discusses stigma and discrimination related to HIV and to HIV risk groups.*

A frame is stigma and discrimination if it does one **or** more of the following:

- It talks about stigma related to HIV
- It gives an example of stigma related to HIV
- It talks about discrimination related to HIV
- It gives an example related to discriminated related to HIV
- It refers to HIV as a taboo

**SOCIAL CAUSES OF HIV FRAME**

*This frame discusses the non-medical causes of HIV*

It discusses the causes of HIV in non medical terms
